# Efficacy of Mobile-Based Cognitive Behavioral Therapy on Lowering Low-density Lipoprotein Cholesterol Levels in Patients With Atherosclerotic Cardiovascular Disease: Multicenter, Prospective Randomized Controlled Trial

**DOI:** 10.2196/44939

**Published:** 2023-04-12

**Authors:** DuanBin Li, Tian Xu, DaQi Xie, MiaoYun Wang, ShuPing Sun, Min Wang, SiSi Zhang, XinRui Yang, ZhongNan Zhang, Shen Wang, Ming Kuang, Jia Tang, HongYing Liu, XuLin Hong, GuoSheng Fu, WenBin Zhang

**Affiliations:** 1 Sir Run Run Shaw Hospital Zhejiang University School of Medicine Hangzhou China; 2 Key Laboratory of Cardiovascular Intervention and Regenerative Medicine of Zhejiang Province Hangzhou China; 3 Ningbo First Hospital Ningbo China; 4 Ningbo Ninth Hospital Ningbo China; 5 Hangzhou Medical College Affiliated Lin An People's Hospital Hangzhou China; 6 Linping Integrative Medicine Hospital Hangzhou China; 7 Zhejiang Greentown Cardiovascular Hospital Hangzhou China; 8 Hangzhou Kang Ming Information Technology Co., Ltd Hangzhou China

**Keywords:** mobile-based cognitive behavioral therapy, low-density lipoprotein cholesterol, atherosclerotic cardiovascular disease, self-efficacy, quality of life

## Abstract

**Background:**

Elevated low-density lipoprotein cholesterol (LDL-C) is an established risk factor for atherosclerotic cardiovascular disease (ASCVD). However, low adherence to medication and lifestyle management has limited the benefits of lowering lipid levels. Cognitive behavioral therapy (CBT) has been proposed as a promising solution.

**Objective:**

This trial aimed to evaluate the efficacy of mobile-based CBT interventions in lowering LDL-C levels in patients with ASCVD.

**Methods:**

This multicenter, prospective, randomized controlled trial enrolled 300 patients with ASCVD, who were randomly assigned to the mobile-based CBT intervention group and the control group in a ratio of 1:1. The intervention group received CBT for ASCVD lifestyle interventions delivered by WeChat MiniApp: “CBT ASCVD.” The control group only received routine health education during each follow-up. The linear regression and logistic regression analyses were used to determine the effects of a mobile-based CBT intervention on LDL-C, triglyceride, C-reactive protein, the score of General Self-Efficacy Scale (GSE), quality of life index (QL-index), and LDL-C up-to-standard rate (<1.8 mmol/L) at the first, third, and sixth months.

**Results:**

Finally, 296 participants completed the 6-month follow-up (CBT group: n=148; control group: n=148). At baseline, the mean LDL-C level was 2.48 (SD 0.90) mmol/L, and the LDL-C up-to-standard rate (<1.8 mmol/L) was 21.3%. Mobile-based CBT intervention significantly increased the reduction of LDL-C change (%) at the 6-month follow-up (β=–10.026, 95% CI –18.111 to –1.940). In addition, this benefit remained when baseline LDL-C <1.8 mmol/L (β=–24.103, 95% CI –43.110 to –5.095). Logistic regression analysis showed that mobile-based CBT intervention moderately increased the LDL-C up-to-standard rates (<1.8 mmol/L) in the sixth month (odds ratio 1.579, 95% CI 0.994-2.508). For GSE and QL-index, mobile-based CBT intervention significantly increased the change of scores (%) at the 1-, 3-, and 6-month follow-up (all *P* values <.05).

**Conclusions:**

In patients with ASCVD, mobile-based CBT is effective in reducing LDL-C levels (even for those who already had a standard LDL-C) and can improve self-efficacy and quality of life.

**Trial Registration:**

Chinese Clinical Trial Registry ChiCTR2100046775; https://www.chictr.org.cn/showproj.aspx?proj=127140

## Introduction

Atherosclerotic cardiovascular disease (ASCVD) remains a major global cause of morbidity and mortality, which leads to a substantial economic burden [1,2]. In the United States, the direct and indirect annual cost of ASCVD was estimated to be US $378.0 billion from 2017 to 2018 [3]. The fact that elevated levels of low-density lipoprotein cholesterol (LDL-C) were established risk factors for ASCVD is well accepted, given the results of numerous epidemiological and genetic studies, as well as randomized controlled trials (RCTs) [4-6]. Each 1.0 mmol/L absolute reduction in LDL-C is associated with a 20% reduction in the risk of cardiovascular events and a 12% reduction in the risk of death from vascular disease. The greater the absolute reductions of LDL-C, the more significant the proportion of risk reduction [7]. However, even with the emerging development of various lipid-lowering therapies, the attainment rate of LDL-C remains suboptimal [8]. Among people with established ASCVD and high risk of ASCVD, only 26.6% and 42.9%, respectively, achieved LDL-C control targets in a large national cross-sectional study in China [9]. Poor adherence to pharmacotherapies and nonpharmacotherapies might be a major concern for the efficacy of current treatments [10,11]. Improving the population’s awareness of the disease is an integral part of a comprehensive approach to ASCVD management. Despite the importance of the issue, little attention has been paid by health researchers.

Cognitive behavioral therapy (CBT) is one psychological treatment method that emphasizes improving self-efficacy based on a combination of cognitive and behavioral approaches, which will benefit the patients in clinical outcomes [12]. CBT is mainly used to treat anxiety, depression, and mental diseases [13] and can significantly improve mental and psychological symptoms such as anxiety, depression, and hopelessness [14-16]. CBT programs are now gradually being promoted and used to encourage proactive self-management of various chronic health conditions [17-19]. It has been proved that CBT can help patients manage blood glucose levels and body weight [20,21]. With the growing proliferation of smartphones, mobile-based CBT emerged. By improving patient’s behaviors, mobile-based CBT is cost-effective in the management of chronic diseases [22,23]. Given the large number of patients with ASCVD, mobile-based CBT may be economically promising.

This RCT aimed to evaluate the efficacy of mobile-based CBT interventions in improving LDL-C levels among patients with ASCVD.

## Methods

### Study Design

**Figure 1:**
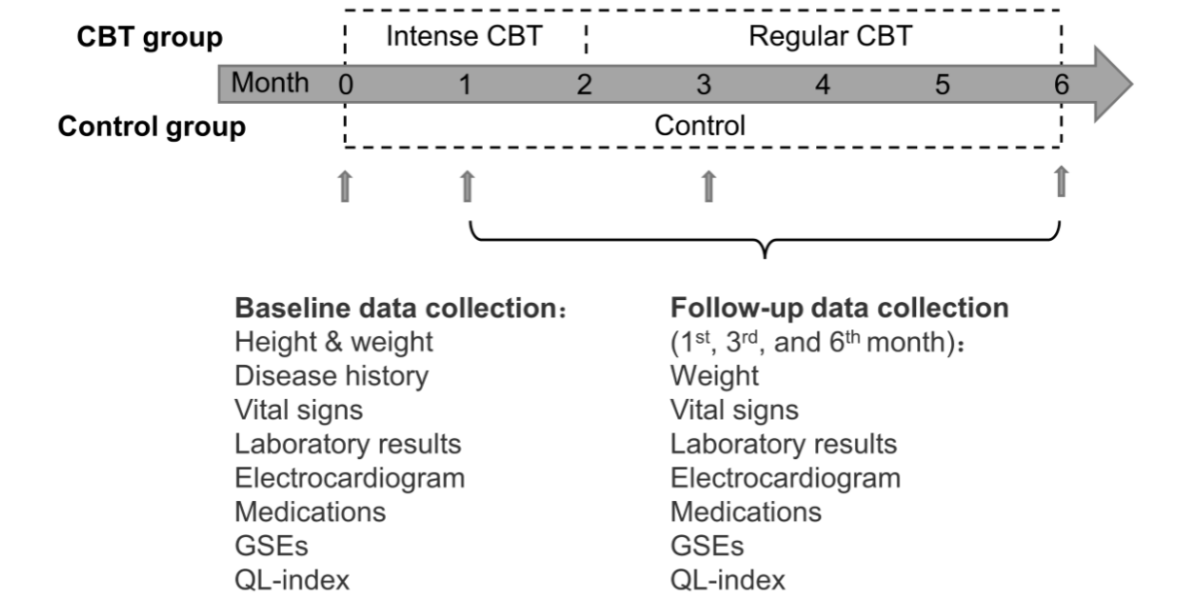
The design of the current randomized controlled trial. CBT: cognitive behavioral therapy; GSE: General Self-Efficacy Scale; QL-index: quality of life index.

This was a prospective, multicenter, and two-arm (allocation ratio was 1:1) trial. It was a nonblinded RCT registered at the Chinese Clinical Trial Registry with the study number ChiCTR2100046775. The design of the current RCT is shown in Figure 1. Participants were recruited from Sir Run Run Shaw Hospital Affiliated to Zhejiang University, Hangzhou, Zhejiang, China; Ningbo Ninth Hospital, Ningbo, China; Zhejiang Xinhua Hospital, Hangzhou, Zhejiang, China; Lin An People Hospital, Hangzhou, Zhejiang, China; and YuHang Integrative Medicine Hospital, Hangzhou, Zhejiang, China through doctors’ face-to-face assessments from June 2021 until December 2022. For more information about centers, see Table S1 in Multimedia Appendix 1.

### Inclusion and Exclusion Criteria

The participants were recruited from among consecutive patients diagnosed with ASCVD. The inclusion criteria were as follows: (1) age from 18 to 80 years; (2) meeting ASCVD diagnostic criteria, including a history of acute coronary syndrome (ACS), myocardial infarction, stable or unstable angina, transient ischemic attack, surgical history of coronary or peripheral vascular reconstructions, etc; (3) currently taking statins by prescription; (4) no difficulty in using smartphones, essential Chinese reading and writing skills and basic calculation skills; and (5) acceptance of the terms and conditions of the study and signature of the informed consent form.

The exclusion criteria were as follows: (1) meeting super high-risk ASCVD diagnostic criteria (diagnostic criteria are determined according to the 2020 Chinese expert consensus on lipid management of very high-risk patients with ASCVD; according to the diagnostic criteria, diagnosed ASCVD patients with one of the following conditions can be diagnosed as super high-risk ASCVD: ASCVD event recurrence; multivessel disease of coronary artery; recent ACS; atherosclerotic vascular disease of heart, brain, or peripheral multivascular bed; LDL-C≥4.9 mmol/L [190 mg/dl]; and diabetes); (2) one or more of the following complications: heart failure, ventricular tachycardia, second- to third-degree atrioventricular block, uncontrolled atrial tachycardia (including atrial flutter, atrial fibrillation, etc), mural thrombus, cardiac aneurysm, dysfunction or rupture of papillary muscle, severe sinus bradycardia (heart rate <50 beats/min), sinus arrest, etc; (3) history of cardiac arrest; (4) acute noncardiogenic complications, such as infections, kidney failure, hyperthyroidism, etc; (5) severe cardiovascular conditions: moderate to severe valvular stenosis, hypertrophic cardiomyopathy, other forms of ventricular outflow tract stenosis, acute myocarditis, pericarditis, active endocarditis, suspected or known aneurysm rupture, acute pulmonary embolism, or pulmonary infarction; and (6) vulnerable populations, including patients with mental disorders, critical illness, age <18 years, pregnancy, students or subordinate of principal investigators, employees of the research institute, etc.

### Sample Size

A power calculation based on an absolute reduction of 20% [24,25] in LDL-C from the baseline expected during 6 months of follow-up indicated that 300 cases were determined as the target sample size for 80% statistical power.

### Ethics Approval

Before conducting the study, written informed consent was obtained from all participants included in the study. The study was carried out according to the Declaration of Helsinki and was approved by the Ethics Committee of Sir Run Run Shaw Hospital (NO.20210508-30). The CONSORT-EHEALTH (Consolidated Standards for Reporting Trials of Electronic and Mobile Health Applications and Online Telehealth) checklist was adhered to report the current study [26].

### Intervention and Control

The subjects were randomly assigned to the intervention group and the control group in a ratio of 1:1 through the electronic data capture (EDC) Trial Data system [27]. The intervention group accessed the WeChat MiniApp by scanning the specified QR code and bound it to WeChat. The background of the applet will review this information to ensure that the patient is a participant in the study. After the audit is passed, the patient will have access to the relevant information in the applet (clock in, video, image, text, etc).

The intervention group received CBT for ASCVD lifestyle interventions delivered by WeChat MiniApp: “CBT ASCVD” (developed by Hangzhou Kang Ming Health Consulting Co., Ltd). The MiniApp user interface (Figure S1 in Multimedia Appendix 1) and the user manual (Figure S2 in Multimedia Appendix 1) are provided. The CBT intervention consists of a continuous cycle of educational topics. A cycle of topics includes 3 sections (Section A: Overview of CBT and Lipids; Section B: Strategies for Lipid Management; and Section C: Case Study; Table S2 in Multimedia Appendix 1). Sections A and C each have 1 video session. Section B contains 4 video sessions on lipids knowledge, lipids with diets, lipids with exercise, and lipids with emotion, respectively. A total of 6 topics constitute a cycle. The corresponding videos are produced on the basis of these topics and arranged to a 6-month intervention period according to the CBT schedule (Figure S3 in Multimedia Appendix 1). The CBT schedule was divided into an intense intervention period (1-2 months) and a regular intervention period (3-6 months). Compared to the regular intervention period, the intense intervention period had more frequent CBT interaction. The contents were designed and reviewed by cardiologists and psychiatrists from Sir Run Run Shaw Hospital, Zhejiang University, Hangzhou, Zhejiang, China. This WeChat MiniApp does not limit the number of login times of patients in the intervention group, but patients need to complete the corresponding courses every week according to the learning plan; otherwise, they will receive SMS text message reminders.

The design of this content is intended to improve the cognition and behavior of patients on blood lipid management through CBT and improve the self-management level (self-efficacy). Long-term accumulation will help to change the outcome of blood lipid management so that the blood lipid level of patients can not only meet the treatment goal but also be stable for a long time. The “CBT ASCVD” can systematically interfere with patients’ cognition, emotion, and behavior through mobile terminals. Participants in the intervention group have been told how to access the program after registration and will use it during the study.

In addition, routine care was delivered to both control group and CBT group. Routine care was designed as a brief oral health education by healthcare providers during each follow-up, involving disease-related knowledge, lifestyle guidance, complication prevention, rational drug use, and other contents. Besides, all participants received standard pharmacological treatments.

### Data Collection and Assessment

The EDC system was used for data collection and management. Data collection occurred at 4 points: baseline, follow-up in the first, third, and sixth months. Baseline data were collected when patients were enrolled, including height, weight, disease history, vital signs, laboratory results, electrocardiogram, medications, General Self-Efficacy Scale (GSE), and quality of life index (QL-index). Follow-up data were collected at the follow-up in the first, third, and sixth months, including weight, vital signs, laboratory results, electrocardiogram, medications, GSE, and QL-index (Figure 1).

All laboratory test samples were obtained after an overnight fast. Triglyceride (TG) level was measured by the standard enzymatic method. The Friedewald formula calculated the LDL-C level [28]. The definition of variability is as follows: (1) SD method SD is used to describe the variability of the univariate during the follow-up period; (2) coefficient of variation (CV) method: CV = (SD / mean) × 100(%) [29]. The LDL-C standard was defined as LDL-C below 1.8 mmol/L [30].

The GSE is a psychometric scale that assesses self-efficacy [31]. It consists of 10 items that represent attitudes toward obstacles rated on a scale from 0 (not optimal) to 4 (very optimal). The QL-index is a tool to evaluate the QL [32], consisting of activities of daily living, principal activities, health, outlook, and support.

### Outcomes

Given routine clinical practice and the time required to achieve maximal lipid reduction, the follow-up period was designed to be 6 months from the start of lipid-lowering medication, which was concurrent with the CBT intervention. The study’s primary outcome was the change in the LDL-C level from enrollment to each follow-up node. The secondary outcomes were TG, C-reactive protein (CRP), the score of GSE, and the QL-index.

### Statistical Analysis

The normally distributed continuous variable was presented as mean (SD) with comparisons by independent samples *t* tests. The nonnormally distributed continuous variable was presented as median (IQR) with comparisons by the Kruskal-Wallis test. The categorical variable was presented as a count (percentage) with comparisons by the chi-square test.

The distribution of indicators (LDL-C, TG, QL-index, and GSE) between the control and the CBT group in the sixth month was visualized by the violin plot and compared by the Kruskal-Wallis test. The trajectory of indicators during the follow-up period was visualized according to the control and CBT groups. The linear regression model was used to assess the association of CBT with the change in LDL-C and the variation of LDL-C, TG, CRP, and the score of QL-index and GSE. The logistic regression model was used to assess the association between CBT and LDL-C during the follow-up period. We additionally adjusted this rate at baseline in the adjusted model. The subgroup analyses were conducted for the change of LDL-C, QL-index, and GSE at the sixth month stratified by the baseline LDL-C (<1.8 or ≥1.8 mmol/L), gender (male or female), age (<65 or ≥65 years), hypertension (no or yes), and diabetes (no or yes).

## Results

### Study Participants

**Figure 2:**
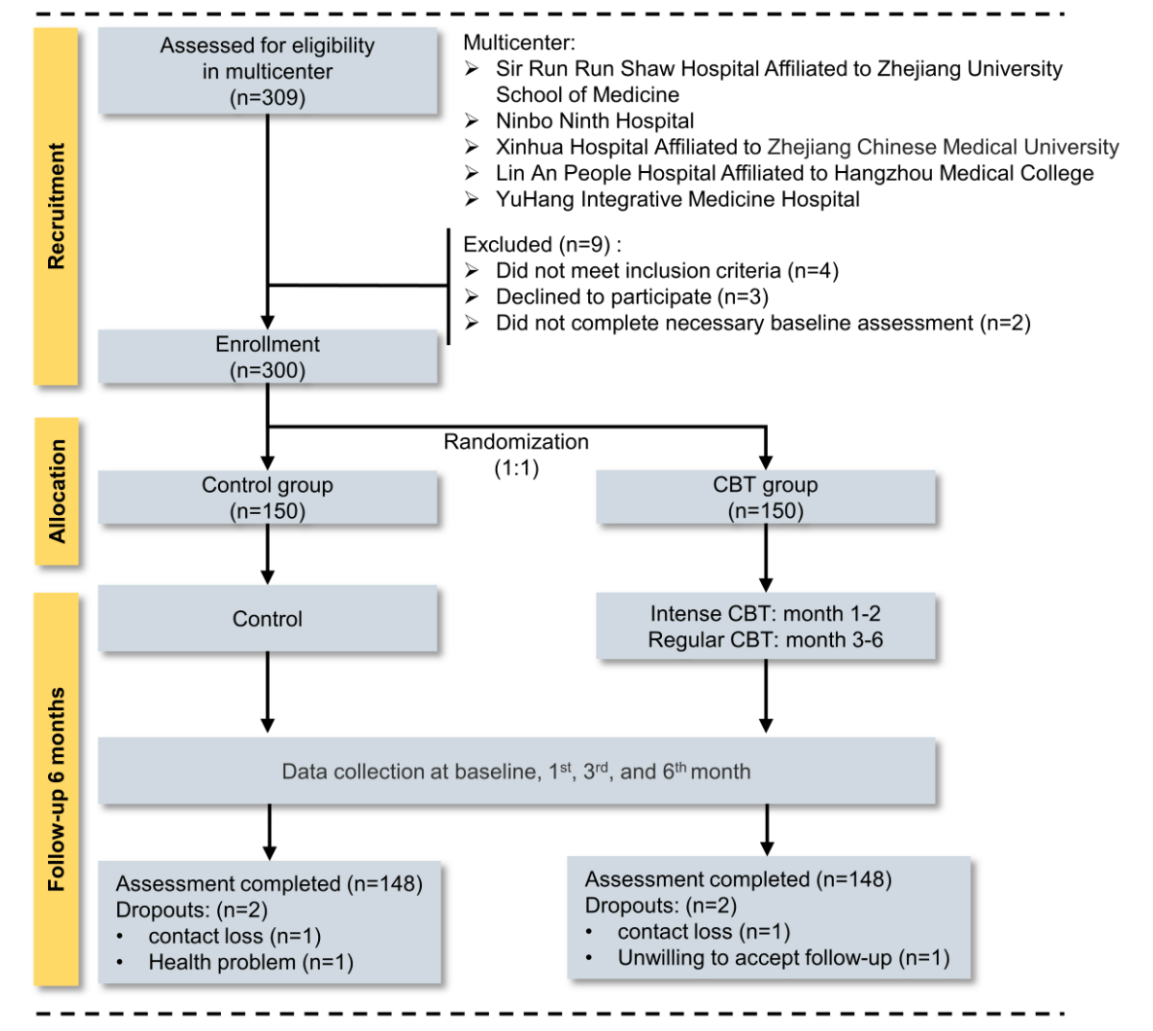
Flow diagram of patient recruitment. CBT: cognitive behavioral therapy.

A total of 300 patients were enrolled and randomly allocated to the control group (n=150) and the CBT group (n=150). Overall, 296 patients completed assessments during the 6-month follow-up. In the control group, 148 patients completed the assesments, and 2 patients dropped out due to contact loss (n=1) and health problems (n=1). In the CBT group, 148 patients completed, and 2 patients dropped out due to contact loss (n=1) and unwillingness to accept follow-up (n=1). Figure 2 presents the flowchart of patient recruitment.

### Baseline Characteristics

The baseline characteristics are displayed in Table 1. The mean age was 63.04 (SD 9.69) years, 69.6% were male participants, and the mean LDL-C level was 2.48 (SD 0.90) mmol/L, and 21.3% up-to-standard rates for LDL-C. There were no significant differences in baseline characteristics by group condition (CBT group vs control group), including demographics (age, gender, BMI, smoking, and drinking), comorbidity (diabetes and hypertension), laboratory results (LDL-C, TG, CRP), LDL-C <1.8 mmol/L, LDL-C <1.8 mmol/L and TG <5.6 mmol/L, GSE score and QL-index (all *P* values >.05).

**Table 1:** Baseline characteristics.

Characteristics		Overall (N=296)	Control (n=148)	CBT^a^ (n=148)	*P* value	
Age (years), mean (SD)		63.04 (9.69)	63.02 (9.45)	63.06 (9.96)	.97	
Male, n (%)		206 (69.6)	102 (68.9)	104 (70.3)	.90	
**Smoke, n (%)**						.94
	Current	36 (12.2)	19 (12.8)	17 (11.5)		
	Ever	38 (12.8)	19 (12.8)	19 (12.8)		
	Never	222 (75.0)	110 (74.3)	112 (75.7)		
**Drink, n (%)**						.07
	Current	44 (14.9)	29 (19.6)	15 (10.1)		
	Ever	15 (5.1)	7 (4.7)	8 (5.4)		
	Never	237 (80.1)	112 (75.7)	125 (84.5)		
BMI (kg/m^2^), mean (SD)		24.30 (3.56)	24.44 (3.65)	24.17 (3.47)	.52	
Hypertension, n (%)		190 (64.2)	88 (59.5)	102 (68.9)	.12	
Diabetes, n (%)		75 (25.3)	39 (26.4)	36 (24.3)	.79	
LDL-C^b^ (mmol/L), median (IQR)		2.27 (1.88-2.96)	2.28 (1.97-2.96)	2.24 (1.79-2.96)	.40	
LDL-C <1.8 mmol/L, n (%)		63 (21.3)	25 (16.9)	38 (25.7)	.09	
LDL-C <1.8 mmol/L and TG^c^ <5.65 mmol/L, n (%)		62 (20.9)	25 (16.9)	37 (25.0)	.12	
TG (mmol/L), median (IQR)		1.39 (1.03-2.00)	1.40 (1.09-2.08)	1.38 (0.98-1.85)	.23	
CRP^d^ (mg/L), median (IQR)		0.80 (0.40-2.42)	0.90 (0.40-2.62)	0.80 (0.40-2.00)	.38	
QL-index^e^ score, mean (SD)		6.09 (2.42)	6.26 (2.44)	5.93 (2.40)	.24	
GSE^f^ score, mean (SD)		1.90 (0.70)	1.90 (0.70)	1.91 (0.71)	.93	

^a^CBT: cognitive behavioral therapy.

^b^LDL-C: low-density lipoprotein cholesterol.

^c^TG: triglyceride.

^d^CRP: C-reactive protein.

^e^QL-index: quality of life index.

^f^GSE: General Self-Efficacy Scale.

### Changes in LDL-C, TG, GSE, and QL-Index During Follow-up

**Figure 3:**
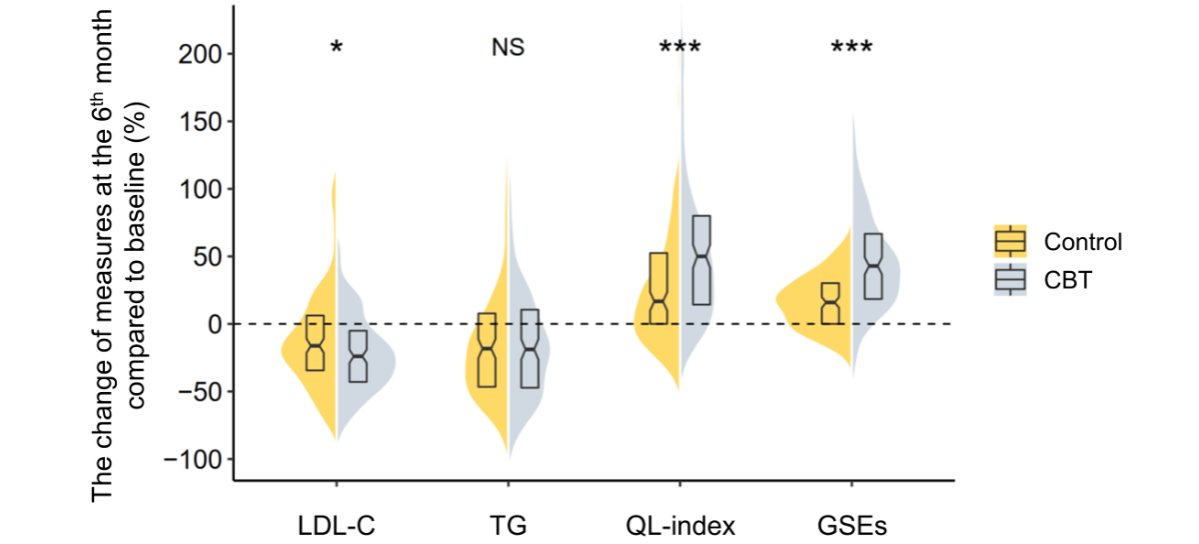
The violin plot for the distribution of each indicator between the control and CBT group. The horizontal axis displayed indicators including LDL-C, TG, QL-index, and GSE. The vertical axis represented the increase/decrease ratio of indicators at the sixth month’s follow-up compared to baseline. The yellow shadow area represented the whole distribution of control group, whereas the gray shadow area represented the whole distribution of the mobile-based CBT intervention group. Each half of violin plot contained a boxplot of the data. The middle short horizontal lines were medians and the black rectangles represented interquartile range. The Kruskal-Wallis test was used to compare differences between groups. CBT: cognitive behavioral therapy; GSE: General Self-Efficacy Scale; LDL-C: low-density lipoprotein cholesterol; NS: no significance; QL-index: quality of life index; TG: triglyceride. *P<.05, **P<.01, ***P<.001.

After 6 months of follow-up, the CBT intervention group (vs control group) had more LDL-C reduction, higher scores of GSE and QL-index, and no difference in TG decrease (Figure 3).

**Figure 4:**
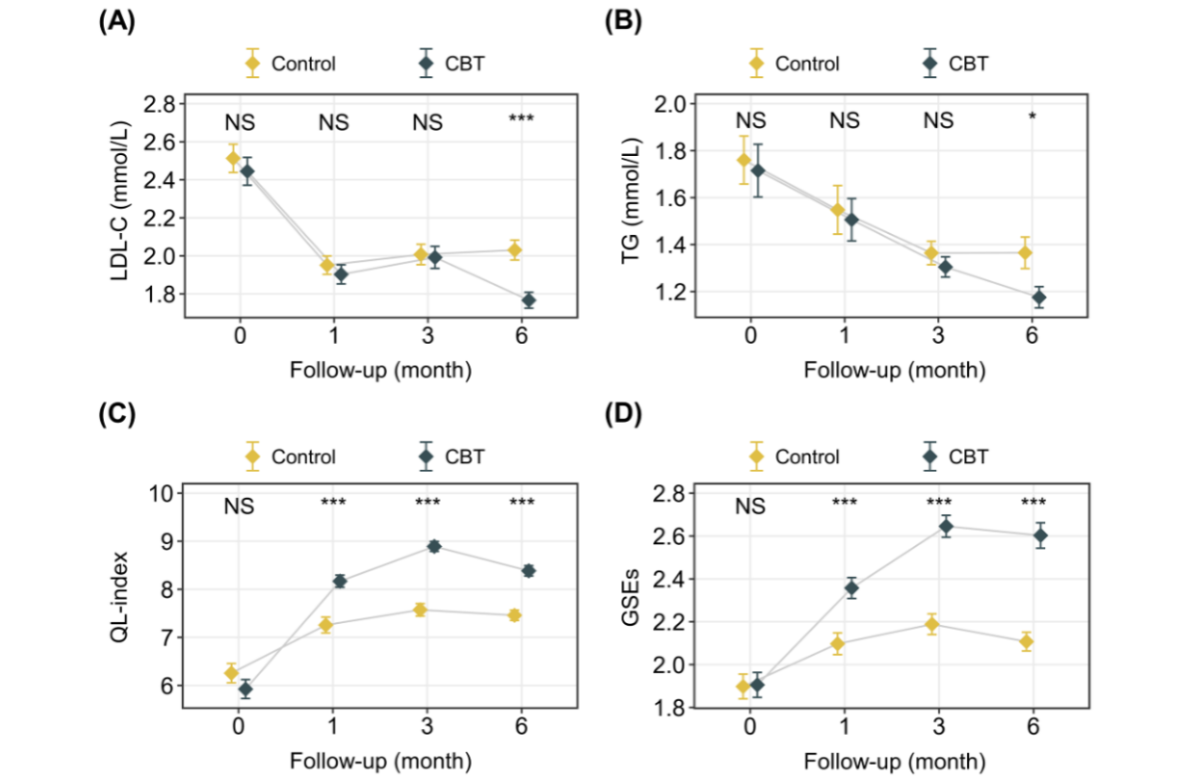
The line chart for the changes in each indicator in the control group and the CBT group. The yellow point indicated the mean of indicator at each time node in the control group. The black point indicated the mean of indicator at each time node in the CBT intervention group. The short horizontal line above and below each point indicated the standard deviation. CBT: cognitive behavioral therapy; GSE: General Self-Efficacy Scale; LDL-C: low-density lipoprotein cholesterol; NS: no significance; QL-index: quality of life index; TG: triglyceride. *P<.05, **P<.01, ***P<.001.

The trajectory of the indicators is visualized in Figure 4. The mean level of LDL-C and TG showed an overall downward trend during the follow-up period in both the control group and the CBT intervention group, and the mean score of QL-index and GSE showed an upward trend.

### Effects of CBT on LDL-C

In Table 2, CBT intervention has no significant effect on the changes in the LDL-C level at the first (β=–1.723, 95% CI –9.762 to 6.315) and third (β=2.641, 95% CI –5.785 to 11.068) months of follow-up. The CBT intervention significantly reduced LDL-C levels compared to the control group at the sixth follow-up month (β=–10.026, 95% CI –18.111 to –1.940). Moreover, a linear regression analysis showed no significant effect on the variation of LDL-C calculated by the SD and the CV method was observed during follow-up (SD: β=–0.018, 95% CI –0.098 to 0.061; CV: β=0.003, 95% CI –0.028 to 0.034).

In Table 3, the CBT intervention has no significant effect on the up-to-standard rates of LDL-C at the first (odds ratio [OR] 1.177, 95% CI 0.745-1.861) and third (OR 0.873, 95% CI 0.553-1.378) months of follow-up. CBT intervention significantly increased the up-to-standard rates of LDL-C at the sixth follow-up month (CBT vs control: 45.3% vs 57.4%; OR 1.631, 95% CI 1.030-2.582). After adjusting the baseline up-to-standard rates of LDL-C, the CBT intervention remained the moderately increased up-to-standard rates (OR 1.579, 95% CI 0.994-2.508).

**Table 2:** Linear regression analyses of cognitive behavioral therapy on LDL-C level.

Variables		β (95% CI)		*P* value
**Changes of LDL-C^a^**				
	First month	–1.723 (–9.762 to 6.315)	.68	
	Third month	2.641 (–5.785 to 11.068)	.54	
	Sixth month	–10.026 (–18.111 to –1.940)	.02^b^	
**Variation of LDL-C**				
	SD	–0.018 (–0.098 to 0.061)	.65	
	CV^c^	0.003 (–0.028 to 0.034)	.85	

^a^LDL-C: low-density lipoprotein cholesterol.

^b^*P* value <.05.

^c^CV: coefficient of variance.

**Table 3:** Logistic regression analyses of CBT on the up-to-standard rates of LDL-C.

Month	LDL-C^a^ <1.8 mmol/L (%)		Crude model, OR (95% CI)	Adjusted model, OR (95% CI)
	Control	CBT^b^		
Baseline	16.9	25.7	—^c^	—
First month	43.9	48.0	1.177 (0.745-1.861)	1.031 (0.635-1.673)
Third month	50.0	46.6	0.873 (0.553-1.378)	0.773 (0.481-1.243)
Sixth month	45.3	57.4	1.631 (1.030-2.582)	1.579 (0.994-2.508)

^a^LDL-C: low-density lipoprotein cholesterol.

^b^CBT: cognitive behavioral therapy.

^c^Not applicable.

### Subgroup Analysis of CBT on LDL-C

**Figure 5:**
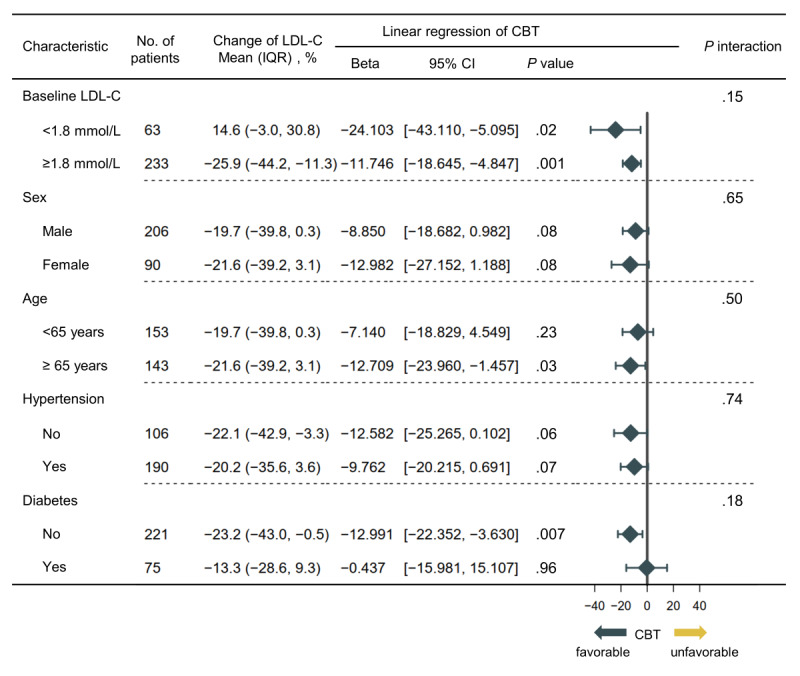
Forest plots of CBT intervention on the changes in LDL-C in prespecified subgroups. Linear regression analyses were conducted to explore the effects of CBT intervention for the changes in LDL-C. CBT: cognitive behavioral therapy; LDL-C: low-density lipoprotein cholesterol.

In Figure 5, the results of the subgroup analysis are consistent with the main finding according to baseline LDL-C (<1.8 or ≥1.8 mmol/L), gender (male or female), age (<65 or ≥65 years), hypertension (yes or no), and diabetes (yes or no). The CBT intervention created a significant reduction in the LDL-C level at the sixth follow-up month in both the baseline LDL-C ≥1.8 mmol/L subgroup (β=–11.746, 95% CI –18.645 to –4.847) and baseline LDL-C <1.8 mmol/L subgroup (β=–24.103, 95% CI –43.110 to –5.095). Hence, the benefits of the CBT intervention were universal. CBT could reduce LDL-C levels even when the baseline LDL-C meets the standard. Besides, the significant reduction in receiving the CBT intervention was also observed in older adults (age ≥65 years; β=–12.709, 95% CI –23.960 to –1.457) and the nondiabetes (β=–12.991, 95% CI –22.352 to –3.630) subgroup.

### Effects of CBT on Other Indicators

In Table 4, patients with ASCVD who received the CBT intervention showed significant improvements in the GSE and QL-index during the whole follow-up period (all *P* values <.05). However, the CBT intervention did not significantly affect TG and CRP levels (all *P* values ≥.05). Furthermore, subgroup analyses also suggest that mobile-based CBT increases GSE (Figure S4 in Multimedia Appendix 1) and QL-index (Figure S5 in Multimedia Appendix 1) at the sixth month across the stratified population.

**Table 4:** Linear regression analyses of cognitive behavioral therapy on other indicators.

Variables^a^			β (95% CI)		*P* value
**First month**					
	TG^b^	1.842 (–17.314 to 20.998)		.85	
	CRP^c^	54,326 (–33,254 to 141,906)		.23	
	QL-index^d^	32.85 (16.026 to 49.666)		<.001^e^	
	GSE^f^	16.96 (11.632 to 22.291)		<.001^e^	
**Third month**					
	TG	0.001 (–12.559 to 12.559)		>.99	
	CRP	96,721 (–91,948 to 285,390)		.32	
	QL-index	38.616 (16.271 to 60.96)		.001^e^	
	GSE	27.088 (19.952 to 34.224)		<.001^e^	
**Sixth month**					
	TG	–10.194 (–22.745 to 2.357)		.11	
	CRP	–10,723 (–62,374 to 40,928)		.68	
	QL-index	24.834 (3.012 to 46.657)		.03^e^	
	GSE	28.199 (21.072 to 35.325)		<.001^e^	

^a^The dependent variable is a continuous variable, which is the percentage change relative to the baseline level.

^b^TG: triglyceride.

^c^CRP: C-reactive protein.

^d^QL-index: quality of life index.

^e^*P*<.05.

^f^GSE: General Self-Efficacy Scale.

## Discussion

### Principal Findings

This multicenter RCT showed that compared with the conventional interventions group, a significant reduction of LDL-C level in the CBT group was observed at the 6-month follow-up. This difference was consistently significant even when the baseline LDL-C had reached the standard. The CBT intervention could significantly improve the self-efficacy and quality of life of patients with ASCVD, which was present during the whole follow-up period.

### Comparison to Prior Work

Controlling lipids and managing chronic conditions represent extensive and costly challenges for patients with ASCVD, which require long-term medication adherence and lifestyle modifications. Moreover, psychological distress can be considered for patients living with a chronic disease [33,34]. Lifestyle changes, such as losing weight, choosing healthy food, exercising, and stopping smoking, are challenging. CBT is devoted to positively changing how a person thinks (“cognitive”) and what they do (“behavior”) [35]. It has been increasingly used to modify cardiovascular risk factors in psychosocial intervention programs [36,37]. When difficulties in controlling lipids occur, patients with cognitive distortions become frustrated and abandon medical interventions, which worsens their condition. CBT can increase the readiness of patients to cope with their problems and help them with their resolution [38,39]. A cardiac rehabilitation program showed that CBT significantly improved depressive symptoms and high-density lipoprotein cholesterol levels [40]. Similarly, another small-scale RCT demonstrated that CBT could reduce LDL-C, TC, and TG levels in patients with diabetes [20]. Consistent with the previous results, this study demonstrated a significant reduction in LDL-C levels in patients with ASCVD after they receive a 6-month CBT invention.

### Mobile-Based CBT and ASCVD

Although the traditional CBT model can provide professional medical interventions during admission, it is limited by location. It cannot achieve timely and accurate medical follow-up once patients leave the hospital. Web-based CBT is as efficacious as face-to-face CBT [41] and can be delivered at a fraction of the cost and clinical time. With the development of the mobile internet, WeChat has built a critical communication platform for social intercourse [42]. In WeChat, we also developed a public account, thus making it easier to communicate between physicians and patients. To ensure data security and privacy, MiniApp does not collect any patient data from the hospital information system, nor does it require patients to upload any medical information to WeChat. MiniApp only serves as a convenient mobile-based CBT platform that works one way from the health care provider to the patient. Despite potential information security issues, WeChat remains one of the preferred platforms for CBT due to its large number of users in mainland China.

CBT controls lipid levels for patients with ASCVD by improving drug compliance, strengthening physical exercise, a balanced diet, and a healthy lifestyle [43-45]. Moreover, CBT-based interventions can help improve lipid profiles by decreasing physiological stress caused by negative emotions and improving autonomic nervous and endocrine systems [46]. The efficacy of this treatment modality might be slow and long-lasting. Patients may gradually benefit from a sustained CBT intervention. As shown by the results of this study, the lipid-lowering effect of CBT is not immediately observable; no differences were observed at follow-up months 1 and 3, and the difference was not significant until follow-up month 6 (β=–10.026, 95% CI –18.111 to –1.940). The requirement of lipid standard for patients with ASCVD is LDL-C below 1.8 mmol/L [30]. The crude model showed that the up-to-standard rate of LDL-C in patients receiving the CBT intervention increased significantly in the sixth month, compared with the control group (OR 1.631, 95% CI 1.030-2.582). Patients may benefit further by extending the CBT intervention beyond the current 6 months. However, this benefit may change after the end of the intervention due to the potential for relapsing into bad behaviors.

The comprehensive evaluation of the efficacy of the intervention should involve objective indicators and subjective perception. Improving self-efficacy is a strategy to help patients achieve and keep treatment goals [47]. The GSE is a psychometric scale that assesses self-efficacy [31]. The QL-index is a tool to evaluate the quality of life [32], another indicator of subjective perception in this study. Gratifyingly, the CBT intervention’s improvement in self-efficacy and quality of life is immediately present and remains persistent.

### Strengths

Our study is the first multicenter, prospective, randomized, controlled, open-label parallel-group superiority trial conducted in a Chinese population with ASCVD to explore the efficacy of mobile-based CBT on reducing LDL-C levels. The results of this study support that CBT should act as a treatment key in the comprehensive management of ASCVD. On the one hand, CBT can effectively reduce LDL-C levels in patients with ASCVD by improving self-efficacy, and the clinical benefit is more significant with the extension of the intervention time. The CBT intervention is expected to reduce the burden of lipid-lowering drugs or even replace them. On the other hand, the quality of life of patients with ASCVD improved by the CBT intervention is instant and effective. The development of mobile technology has substantially reduced the cost of CBT and increased its convenience and accessibility. Given the large number of patients with ASCVD, future work can be devoted to quantifying the economic benefits of mobile-based CBT by conducting cost-effectiveness analyses. Mobile-based CBT remains a promising option in the comprehensive management of patients with ASCVD.

### Limitations

This study has some limitations worth noting. First, due to the nature of psychotherapy studies, subjects and researchers (excluding statisticians) were unblinded. Therefore, they may be subject to bias from treatment expectations. Second, a reduction of LDL-C was shown after the 6-month mobile-based CBT intervention. Extending the duration of the mobile-based CBT beyond 6 months may be helpful to determine the efficacy of CBT on other indicators. Third, the follow-up was terminated after the end of the 6-month CBT intervention. Further assessment of relapsing into bad behaviors was not conducted.

### Conclusions

In patients with ASCVD, mobile-based CBT is effective in reducing LDL-C levels (even for those who are already meet the LDL-C standard) and can improve self-efficacy and quality of life.

## Appendix

Multimedia Appendix 1 Supplementary figures and tables and CBT ASCVD introduction.

Multimedia Appendix 2 CONSORT-eHEALTH checklist (V 1.6.1).

## Abbreviations

ACS: acute coronary syndrome
ASCVD: atherosclerotic cardiovascular disease
CBT: cognitive behavioral therapy
CONSORT-EHEALTH: Consolidated Standards for Reporting Trials of Electronic and Mobile Health Applications and Online Telehealth
CRP: C-reactive protein
CV: coefficient of variation
EDC: electronic data capture
GSE: General Self-Efficacy Scale
LDL-C: low-density lipoprotein cholesterol
OR: odds ratio
QL-index: quality of life index
RCT: randomized controlled trial
TG: triglyceride
