# Supplementary material for: Efficacy of Mobile-Based Cognitive Behavioral Therapy on Lowering Low-density Lipoprotein Cholesterol Levels in Patients With Atherosclerotic Cardiovascular Disease: Multicenter, Prospective Randomized Controlled Trial
Source: J Med Internet Res. 2023 Apr 12;25:e44939. doi: 10.2196/44939 (PMC10134029; doi:10.2196/44939)
Supplement: Multimedia Appendix 1 [file jmir_v25i1e44939_app1.doc]

**Supplementary Materials**

**Figures**

**Figure S1.** The user interface of MiniApp

**Figure S2.** The user manual of MiniApp

**Figure S3.** The 6-month schedule of CBT intervention

**Figure S4.** The subgroup analysis of CBT on GSEs

**Figure S5.** The subgroup analysis of CBT on QL-index

**Tables**

**Table S1.** Information about multicenter

**Table S2.** The content of a CBT intervention cycle

**Others**

WeChat MiniApp: “CBT ASCVD” introduction

**Figure S1.** The user interface of MiniApp


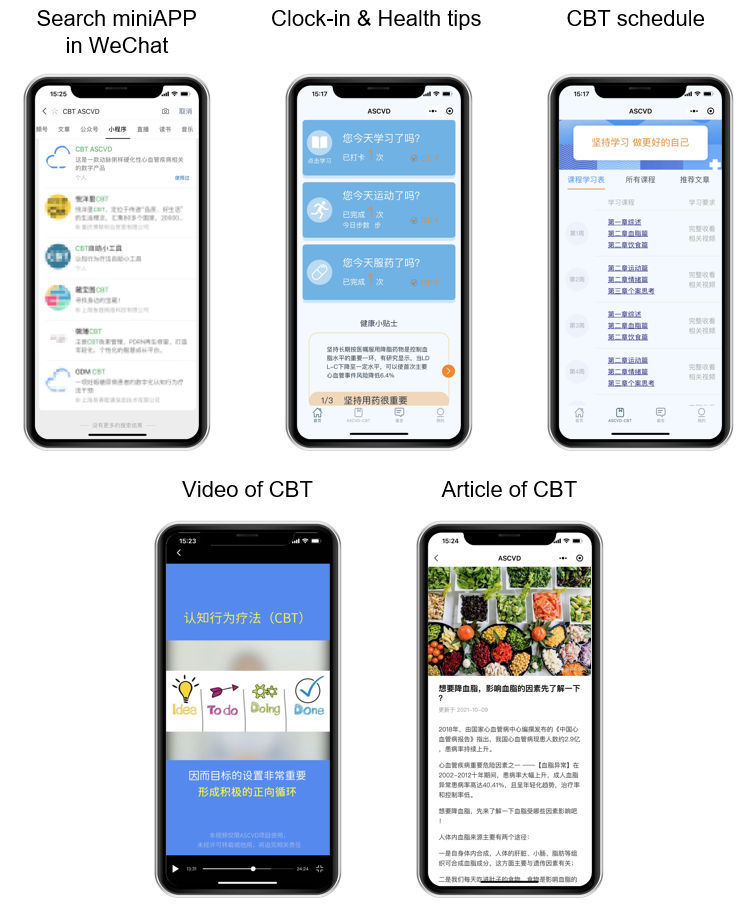


**Figure S2.** The user manual of MiniApp


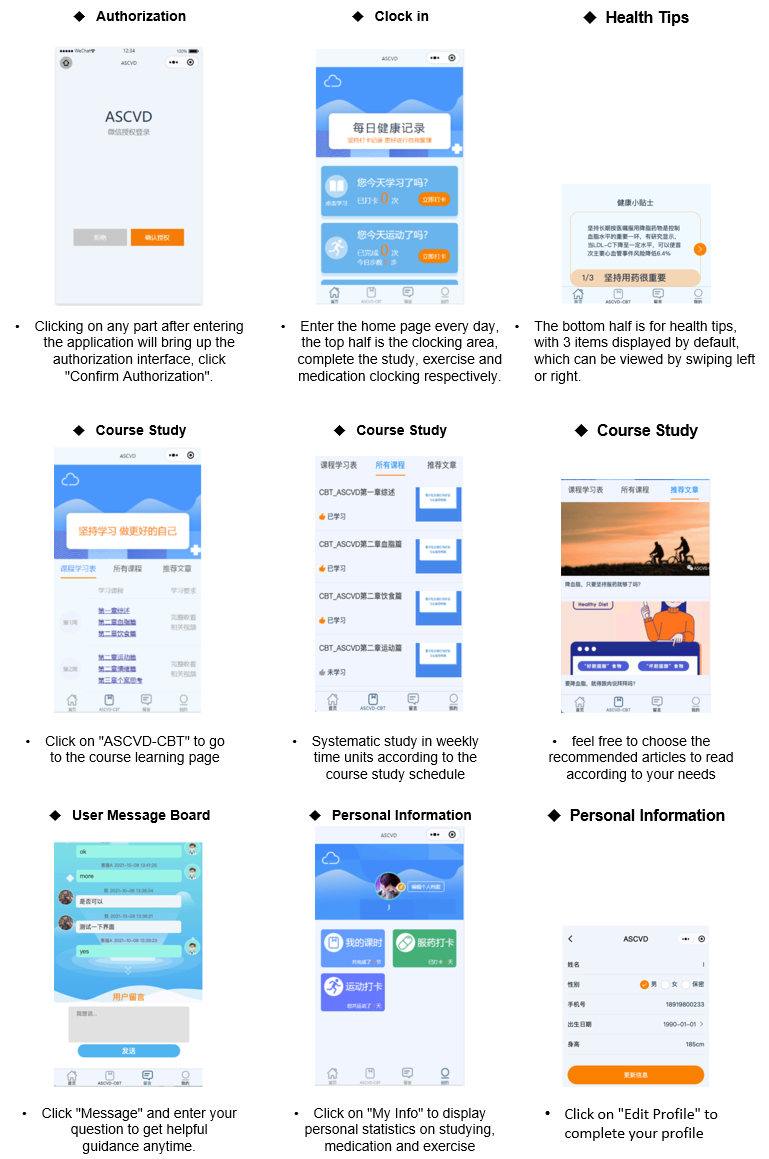


The user manual of MiniApp is provided after translation into English.

**Figure S3.** The 6-month schedule of CBT intervention


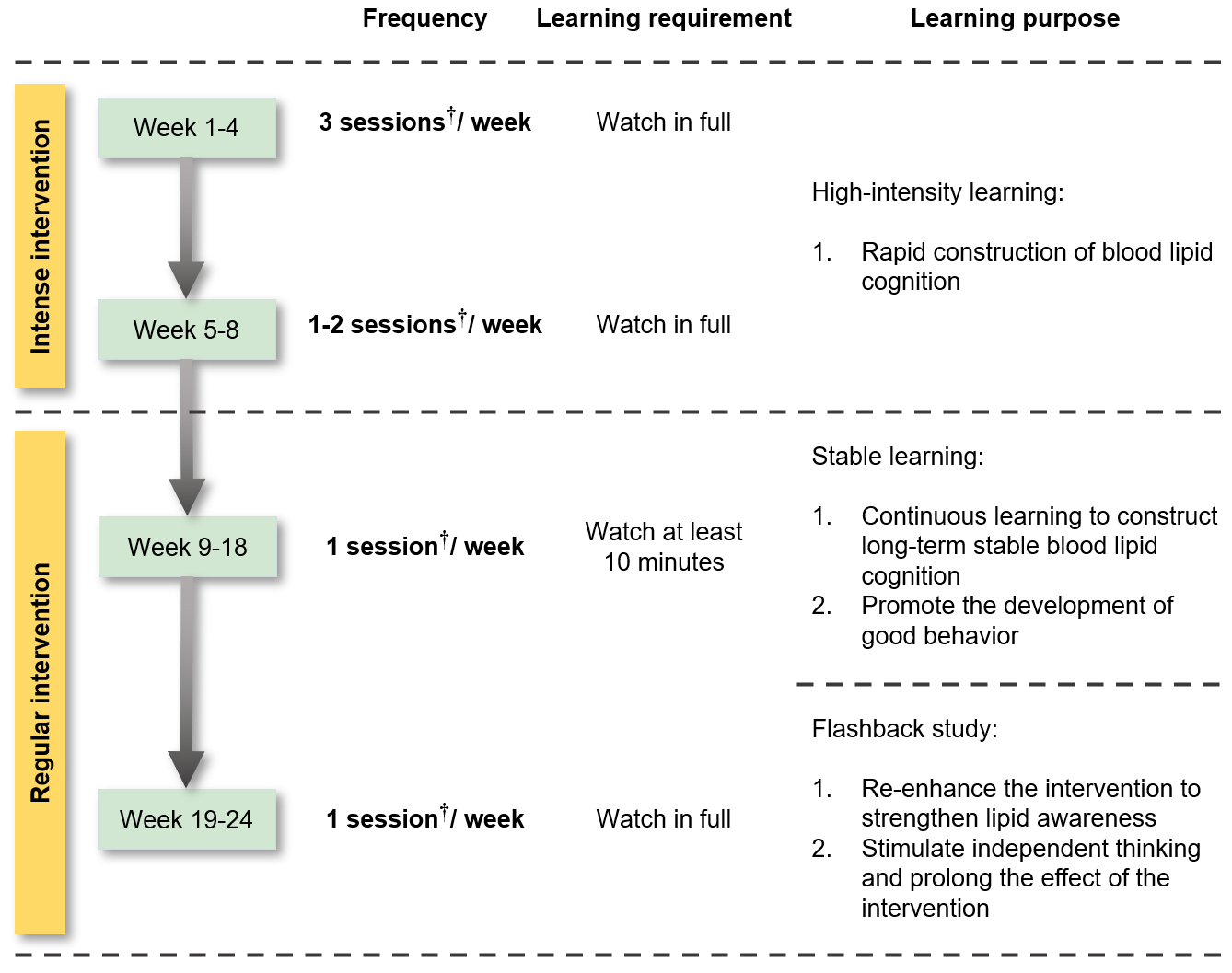


† The cycle of sessions was shown in **Table S2**.

**Figure S4.** The subgroup analysis of CBT on GSEs


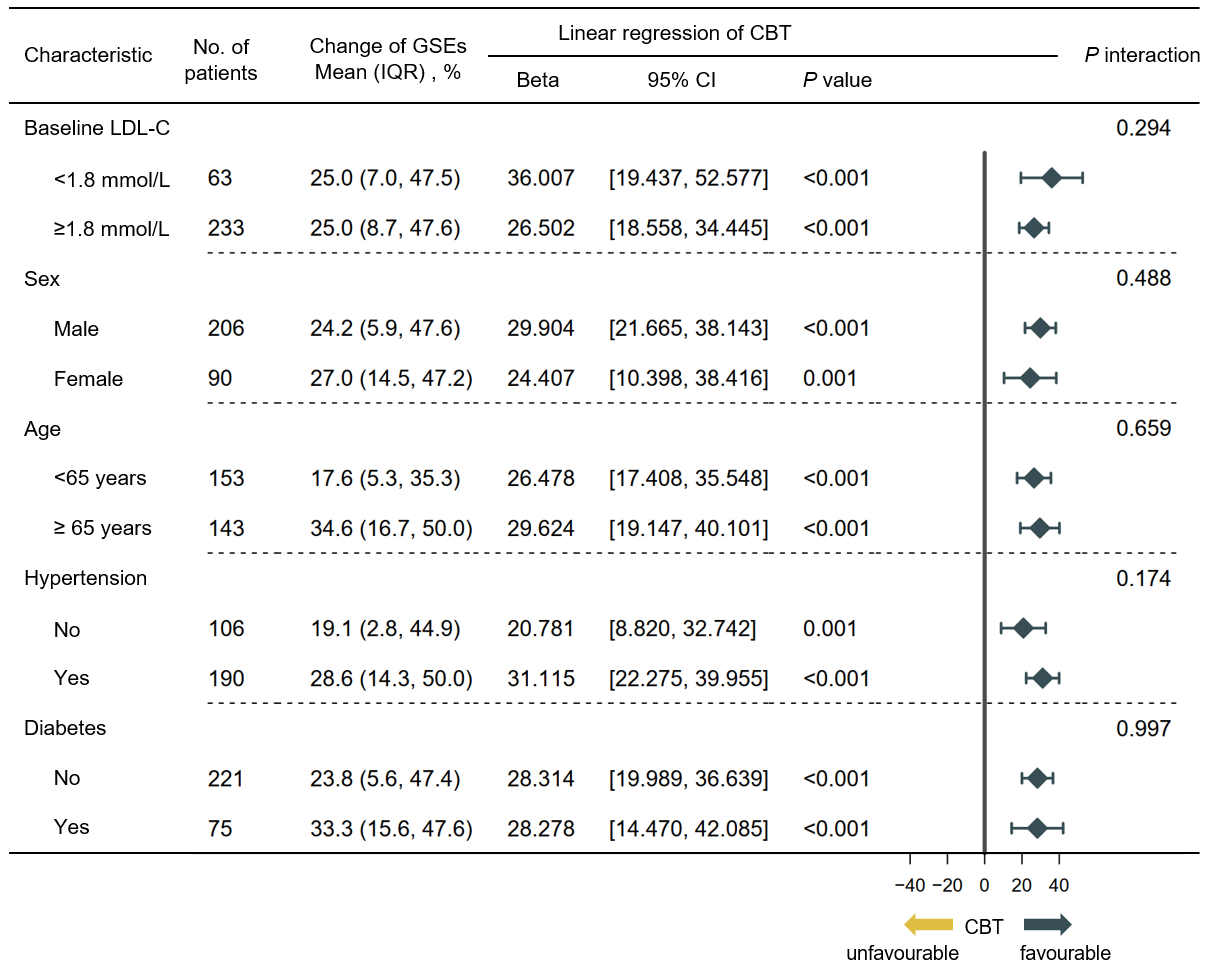


**Figure S5.** The subgroup analysis of CBT on QL-index


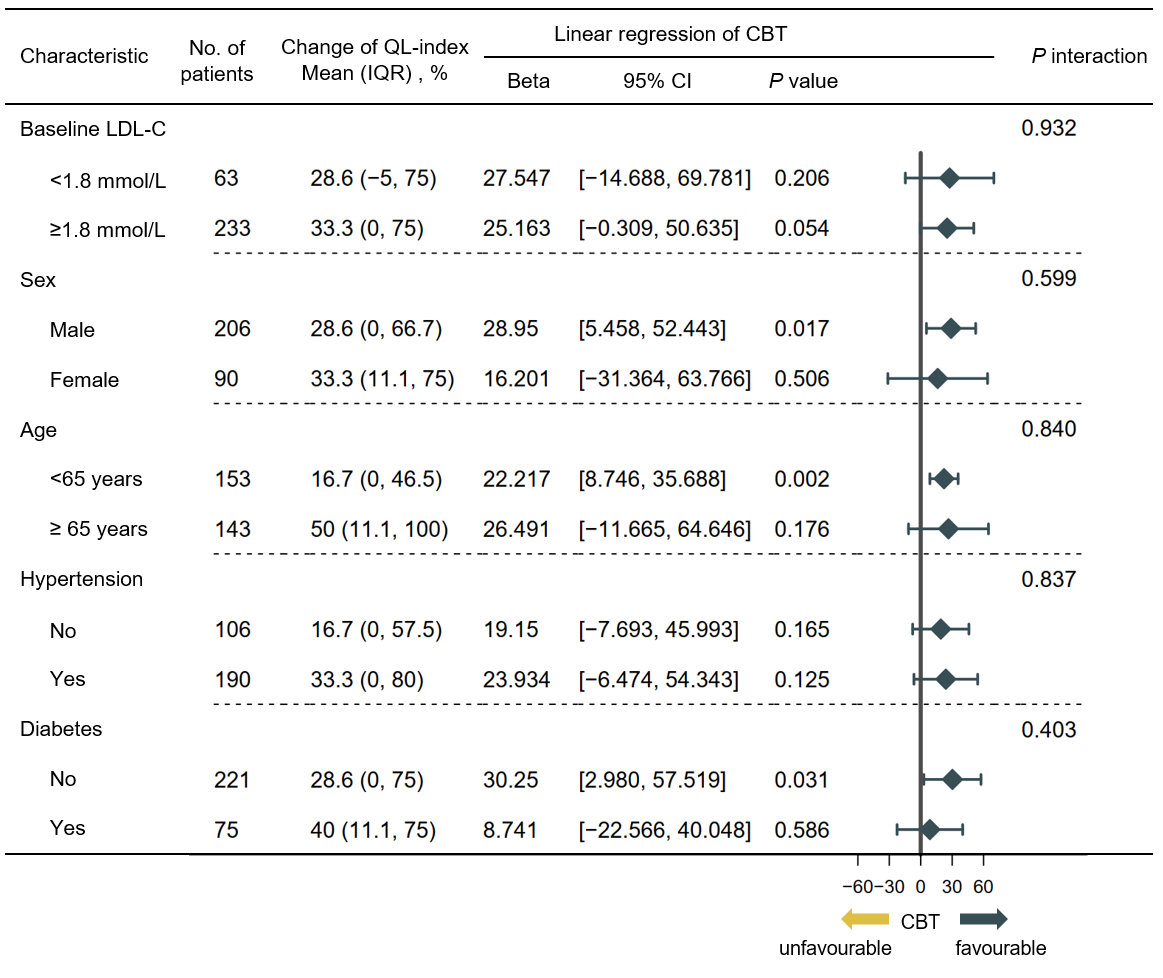


**Table S1.** Information about multicenter

| **Hospital** | **Type** | **University/ College** | **City & Tier** | **Province** | **Country** | **Official website** |
| --- | --- | --- | --- | --- | --- | --- |
| Sir Run Run Shaw Hospital | Teaching hospital | Zhejiang University | Hangzhou,  First-tier city | Zhejiang | China | http://www.srrsh.com/ |
| Ningbo Ninth Hospital | Non-teaching hospital | - | Ningbo,  Second-tier city | Zhejiang | China | http://www.nbsdjyy.com/ |
| Xinhua Hospital | Teaching hospital | Zhejiang Chinese Medical University | Hangzhou,  First-tier city | Zhejiang | China | https://www.xhhos.com/ |
| Lin An people hospital | Teaching hospital | Hangzhou Medical College | Hangzhou,  First-tier city | Zhejiang | China | http://rmyy.lanews.com.cn/ |
| YuHang Integrative Medicine Hospital | Non-teaching hospital | - | Hangzhou,  First-tier city | Zhejiang | China | - |

**Table S2. The content of a CBT intervention cycle**

| **Section** | **Session** |  | **Content** | **Speaker** |
| --- | --- | --- | --- | --- |
| A | 1 |  | **Overview of CBT and lipids:**  1. Introduce the CBT therapy process;  2. Explain the potential effect of CBT therapy on blood lipid management in ASCVD patients;  3. Skills teaching: mindfulness and meditation in CBT. | 1. Clinician (psychiatry);  2. Clinician;  3. Clinician (psychiatry). |
| B | 2 |  | **Lipids knowledge:**  1. The occurrence, development, and clinical significance of blood lipids;  2. Objectives of blood lipid management;  3. Medical methods for blood lipid management: medical treatment and lifestyle change;  4. Importance of medical treatment on blood lipid management;  5. How to achieve high compliance treatment. | 1. Clinician (cardiovascular medicine)  2. Clinician (cardiovascular medicine)  3. Clinician (cardiovascular medicine)  4. Clinician (cardiovascular medicine)  5. Clinician (cardiovascular medicine) |
| B | 3 |  | **Lipids with diet:**  1. High-risk diet (such as high-fat food);  2. Beneficial diet (such as coarse grains);  3. The value of diet control on blood lipid management;  4. How to achieve self-management of diet. | 1. Clinical nutritionist;  2. Clinical nutritionist;  3. Clinical nutritionist;  4. Clinical nutritionist. |
| B | 4 |  | **Lipids with exercise:**  1. High-risk behaviors (such as overweight people who are sedentary);  2. Beneficial exercise (such as more than 45 minutes of moderate-intensity exercise every day);  3. The value of exercise control on blood lipid management;  4. How to achieve self-management in sports. | 1. Clinician (sports medicine);  2. Clinician (sports medicine);  3. Clinician (sports medicine);  4. Clinician (sports medicine). |
| B | 5 |  | **Lipids with emotion:**  1. Bad mood and cardiovascular disease;  2. Positive emotions and cardiovascular diseases;  3. The value of emotional management on blood lipid management;  4. How to achieve self-management of emotions. | 1. Clinician (psychiatry);  2. Clinician (psychiatry);  3. Clinician (psychiatry);  4. Clinician (psychiatry). |
| C | 6 |  | **Case study:**  1. Publicize cases of bad blood lipid management;  2. Guide users' thinking training and promote reflection. | 1. Clinician (cardiovascular medicine);  2. Clinician (cardiovascular medicine). |

The corresponding videos was produced based on these topics and then arranged to 6-month intervention period according to the CBT schedule (refer to **Figure S4.**).

**WeChat MiniApp: “CBT ASCVD” introduction**

**1 Cognitive behavior therapy**

Cognitive behavior therapy (CBT) is a structured, instructional, and goal-oriented psychotherapy, which also has the effect of improving self-efficacy. The level of self-efficacy affects the self-management of patients with chronic diseases, and the quality of self-management affects the therapeutic effect/outcome of chronic diseases. Many studies have shown that CBT has a significant effect on the intervention of healthy living behaviors, including diet, exercise, behavior compliance, substance use disorder (such as tobacco dependence, alcohol dependence), depression, anxiety, etc.

This research plans to intervene in the patient's cognition/behavior on blood lipid management for a long time through the WeChat applet developed with CBT as the methodology, observe the changes in patients' blood lipid levels, whether they can achieve the blood lipid standard and long-term stability, and also observe the changes of patients' self-efficacy level.

**2 APP introduction**

The applet has 4 modules: home page, ASCVD-CBT, message board, personal center.

**2.1 Function introduction of each module**

**2.1.1 Home page**

User behavior record.

1. learning clock-in, 2. sports clock-in, 3. medication clock-in, 4. health tips and other functions.

**2.1.2 ASCVD-CBT**

1. Video course: compulsory (browse one by one according to the weekly course plan; the course duration varies; there will be corresponding marks after the course learning, indicating that the course has been completed this week) 2. Medical image and text: optional (to meet the needs of some users for additional information).

**2.1.3 Message board**

User feedback. Realize single interaction, that is, the interaction between users and the background.

**2.1.4 Personal Center**

The total number of display behavior records; Demonstrate results and inspire user confidence.

In addition, the research also adopted the interaction mode of the WeChat group, which includes doctors, psychologists, nutritionists, and sportsmen. Through the mode of group chat, users' enthusiasm for learning and enthusiasm for cognitive behavior practice are further promoted.
